# Term abdominal pregnancy: a case report

**DOI:** 10.1186/s13256-015-0635-3

**Published:** 2015-07-28

**Authors:** Zelalem Mengistu, Assefa Getachew, Mulat Adefris

**Affiliations:** 1 Department of Obstetrics and Gynecology, University of Gondar, Gondar, Ethiopia; 2 Department of Radiology, University of Gondar, Gondar, Ethiopia

**Keywords:** Abdominal pregnancy, Ectopic pregnancy, Gondar, Term

## Abstract

**Introduction:**

Abdominal pregnancy is a rare form of ectopic pregnancy with very high maternal and fetal morbidity and mortality. A high index of suspicion is crucial for prompt diagnosis and management especially in low-resource countries.

**Case presentation:**

A 32-year-old gravida III para II Amhara woman presented with shortness of breath and progressive abdominal distension and pain. An emergency laparotomy was performed with the impression of abdominal pregnancy. Intraoperatively, the fetus was seen in an intact amniotic sac in her abdomen, her uterus was ruptured at the fundus and the placenta was extensively adhered to the edge of the ruptured site. The patient and neonate progressed well and were discharged.

**Conclusions:**

Term abdominal pregnancy is an extremely rare diagnosis and requires a high index of suspicion. The life-threatening complication is bleeding from the detached placental site. A thorough examination of the newborn is important to rule out congenital anomalies.

## Introduction

Abdominal (peritoneal) ectopic pregnancy (EP), defined as EP occurring within the peritoneal cavity outside genital organs (uterus, tubes, ovaries), represents a very rare form of EP (1% of all EP). Of note, abdominal EP is the only form of EP that can result in a delivery of a healthy full-term baby. The incidence of abdominal pregnancy differs in various publications and ranges between 1:10,000 and 1:30,000 pregnancies. It was first reported in 1708 as an autopsy finding and a number of cases have been reported worldwide with varying presentations [1].

**Fig. 1:**
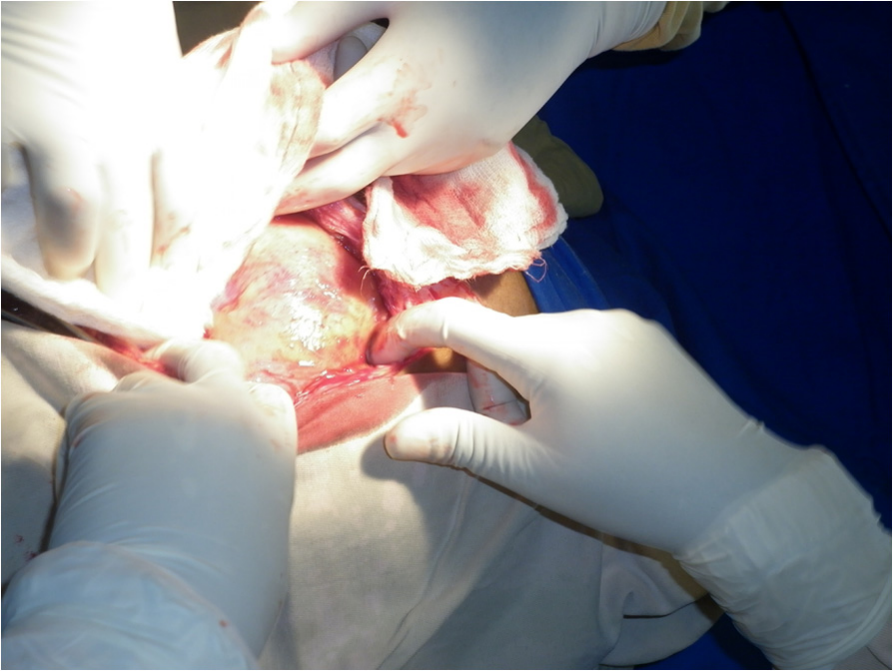
Intact amniotic membrane

**Fig. 2:**
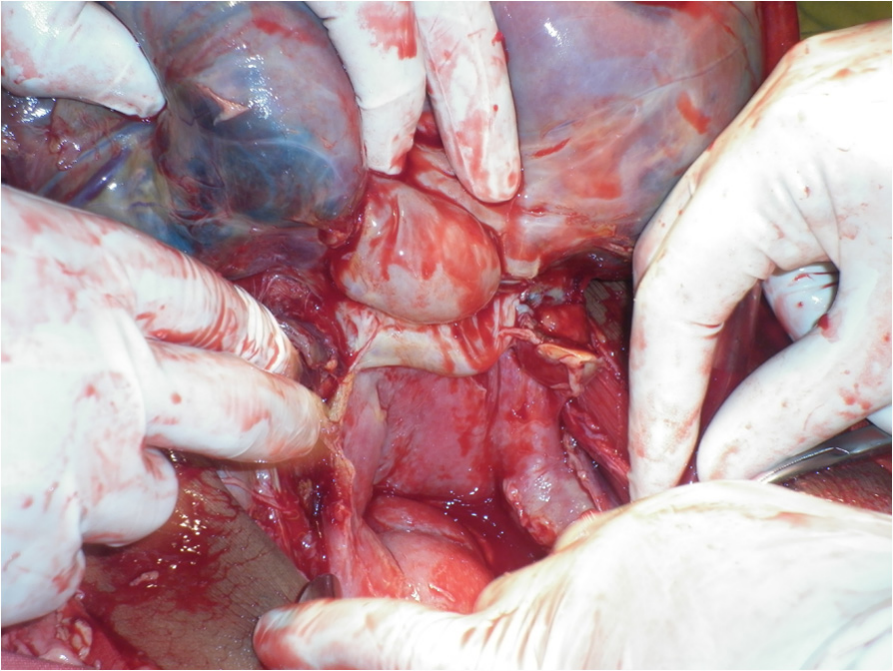
Extensively adherent placenta on serosal surface of the uterus and uterine rupture at the fundus

**Fig. 3:**
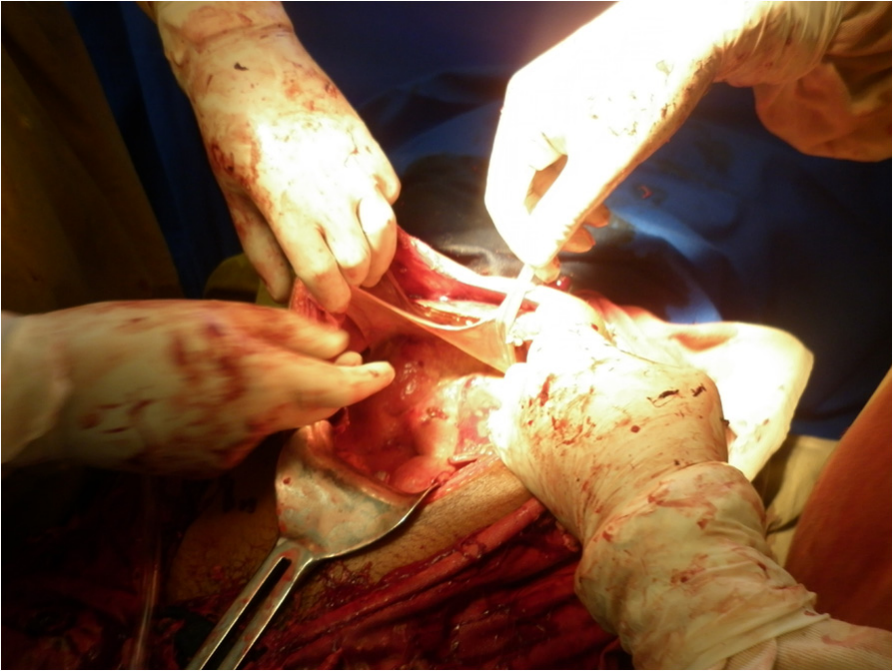
Amniotic membrane attached to the bowel and anterior abdominal wall

Abdominal pregnancy can be classified as primary or secondary. Primary abdominal pregnancy occurs when the fertilized ovum implants directly into the peritoneal cavity; it is the less common type. Secondary abdominal pregnancy occurs when the fertilized ovum first implants in the fallopian tube or uterus, and then due to fimbrial abortion or rupture of the fallopian tube or uterus, the fetus subsequently develops in the mother’s abdominal cavity. Ruptured tubal ectopic pregnancies account for the majority of abdominal pregnancies. Ovarian, tubal and intraligamentary pregnancies are excluded from the definition of abdominal pregnancy [2, 3].

Abdominal pregnancy has a maternal mortality rate between 0.5 and 18% and a perinatal mortality rate between 40 and 95% [4, 5]. We present a rare case of an abdominal pregnancy which reached term with delivery of a live healthy newborn. Both mother and baby were discharged in a good condition.

## Case presentation

Our patient was a 32-year-old gravida III para II Amhara woman who came from a rural area of North West Ethiopia. Her first delivery was an uncomplicated spontaneous vaginal delivery at home at the age of 22 years. Her second delivery was a spontaneous vaginal delivery in a health center 4 years later with early neonatal death for unknown reasons. She was seen by a health professional only once during the current pregnancy, when she experienced progressive abdominal pain 4 months prior to her delivery. She was assessed in a health center and referred to a regional hospital where she underwent an ultrasound scan and was reassured. She presented to the University of Gondar Teaching Hospital with the principal complaint of shortness of breath with associated progressive abdominal distension and pain. Her last menstrual period was unknown but she reported 9 months of amenorrhea.

On general examination, she looked emaciated and was slightly pale. Her vital signs were within normal limits. Her cardiovascular and respiratory system did not reveal any abnormalities. On abdominal examination her symphysis fundal height was term sized, with longitudinal lie and breech presentation. The fetal heart rate was 132 beats per minute and there were no uterine contractions. On vaginal examination the cervix was closed and uneffaced. There was no vaginal bleeding. On ultrasonography examination, there was a singleton live pregnancy with excessive amniotic fluid. The placenta appeared to be attached to the serosal surface of the fundus of her uterus and her uterus was empty. The gestational age was 40 weeks by ultrasound estimation. Her preoperative hematocrit was 34%. An emergency laparotomy was performed with the impression of abdominal pregnancy. Upon opening her abdomen and entering the peritoneum, the fetus was seen in an intact amniotic sac and there was no hemoperitoneum (Fig. 1).

On opening the amniotic sac a live female neonate was delivered weighing 2.6kg. The uterus was ruptured at the fundus and the placenta was extensively adhered to the edge of the ruptured site, the serosal surface of the uterus and to the peritoneum and had infiltrated through the myometrium (Fig. 2).

Her fallopian tubes, ovaries and other abdominal organs were normal. The amniotic sac was attached to her bowels and her anterior abdominal wall and this was removed intraoperatively with no abdominal organ damage. There was significant bleeding after the placenta was detached from her uterus which prompted total abdominal hysterectomy to secure hemostasis and to remove the ruptured uterus. Total estimated intraoperative blood loss was 2L. She was transfused with two units of whole blood intraoperatively and postoperatively and her post-transfusion hematocrit was 27% (Fig. 3).

Her uterus was subjected for histopathology and sections from the infiltrated uterine wall show normal-sized chorionic villi, fibrin and decidual stroma. The patient and neonate progressed well and were discharged.

## Discussion

Advanced abdominal pregnancy (AAP) is classically defined as a pregnancy that has progressed beyond 20 weeks of gestation in which the fetus is growing and developing in the mother’s abdominal cavity, or the fetus shows signs of having been in the mother’s abdominal cavity [6]. It is an extremely rare obstetric complication with high maternal and perinatal mortality. A review of cases from 2008 to 2013 showed that 38 cases of an AAP resulting in a live birth were identified from 16 countries [7].

This case is being reported because no similar case has been reported so far. Abdominal pregnancy could be either primary or secondary to implantation of a primary tubal pregnancy in the peritoneal cavity. The latter is the commonest type [1, 6].

In 1942, Studdiford established three criteria for diagnosis of a primary peritoneal pregnancy: the presence of normal tubes and ovaries, no evidence of uteroperitoneal fistula, and the presence of a pregnancy related exclusively to the peritoneal surface and early enough in gestation to eliminate the possibility of secondary implantation after primary nidation of the tube [2, 3]. Watrowski *et al*. recently expanded the classic Studdiford criteria. They reported a case of an omental pregnancy invading the peritoneum of the Douglas pouch. Thus, secondary peritoneal pregnancy implantation can occur not only after tubal rupture or expulsion of a tubal EP, but also after primary implantation at any other ectopic site [8]. In this case it was clear that the abdominal implantation was secondary based on Studdiford criteria that there is evidence of uteroperitoneal communication.

This case demonstrated how the diagnosis of abdominal pregnancy is difficult and why a high index of suspicion is important in recognizing the condition especially in poorly resourced centers. In this case the diagnosis was missed on initial ultrasound at the regional hospital. Persistent abdominal pain as in this report is the commonest symptom [6]. These features, supported by ultrasonography, made the diagnosis relatively clear in this patient once she appeared at Gondar University Hospital. The diagnosis of abdominal pregnancy is often intraoperative at either diagnostic laparoscopy or laparotomy. Once the condition is suspected, due to fetal malpresentation, malformations or oligohydramnios, then purposeful lateral projection sonography and radiography are helpful. An oxytocin stimulation test and the finding of an abnormally high maternal serum alfa-fetoprotein have been proposed [7]. Other radiological studies such as magnetic resonance imaging and computed tomography scan are helpful in the later stages, unfortunately these advanced imaging technologies are not available in many low-resource countries [1]. The treatment for abdominal pregnancy has traditionally been laparotomy. Recent cases of minimally invasive laparoscopic and ultrasonically guided procedures have emerged in the literature in the last decade for early pregnancy presentations [7, 9].

Bleeding from the placental site can be a life-threatening complication during laparotomy. It is generally recommended to leave the placenta *in situ* and monitor the patient’s human chorionic gonadotropin levels [1, 10]. In this case there was significant bleeding from a detached portion of the placenta and the uterus was ruptured at the fundus, which required total abdominal hysterectomy.

For the newborn, it is very important to rule out congenital malformations. There are reports of fetal malformations as high as 40% associated with abdominal pregnancies [1]. In this case no congenital malformations were detected.

## Conclusions

Term abdominal pregnancy is an extremely rare diagnosis and requires a high index of suspicion. The life-threatening complication is bleeding from the detached placental site. A thorough examination of the newborn is important to rule out congenital anomalies.

## Consent

Written informed consent was obtained from the patient for publication of this case report and accompanying images. A copy of the written consent is available for review by the Editor-in-Chief of this journal.
